# Predictive model for the 5-year survival status of osteosarcoma patients based on the SEER database and XGBoost algorithm

**DOI:** 10.1038/s41598-021-85223-4

**Published:** 2021-03-10

**Authors:** Jiuzhou Jiang, Hao Pan, Mobai Li, Bao Qian, Xianfeng Lin, Shunwu Fan

**Affiliations:** 1 Department of Orthopaedic Surgery, Sir Run Run Shaw Hospital, Medical College of Zhejiang University, Hangzhou, China; 2 Key Laboratory of Musculoskeletal System Degeneration and Regeneration Translational Research of Zhejiang Province, Hangzhou, China; 3 Department of Orthopaedics, The First Affiliated Hospital of Wenzhou Medical University, Wenzhou, China

**Keywords:** Bone cancer, Epidemiology, Outcomes research

## Abstract

Osteosarcoma is the most common bone malignancy, with the highest incidence in children and adolescents. Survival rate prediction is important for improving prognosis and planning therapy. However, there is still no prediction model with a high accuracy rate for osteosarcoma. Therefore, we aimed to construct an artificial intelligence (AI) model for predicting the 5-year survival of osteosarcoma patients by using extreme gradient boosting (XGBoost), a large-scale machine-learning algorithm. We identified cases of osteosarcoma in the Surveillance, Epidemiology, and End Results (SEER) Research Database and excluded substandard samples. The study population was 835 and was divided into the training set (*n* = 668) and validation set (*n* = 167). Characteristics selected via survival analyses were used to construct the model. Receiver operating characteristic (ROC) curve and decision curve analyses were performed to evaluate the prediction. The accuracy of the prediction model was excellent both in the training set (area under the ROC curve [AUC] = 0.977) and the validation set (AUC = 0.911). Decision curve analyses proved the model could be used to support clinical decisions. XGBoost is an effective algorithm for predicting 5-year survival of osteosarcoma patients. Our prediction model had excellent accuracy and is therefore useful in clinical settings.

## Introduction

Osteosarcoma is the most common bone malignancy, with the highest incidence in children and adolescents^1–3^. Osteosarcoma is the eighth most common cancer among childhood cancers^1^. The incidence rate of childhood and adolescent osteosarcoma ranges between 4 and 7 per million persons per year among different ethnicities^1^. The 5-year survival rate is usually used for evaluating treatments or risk factors^1–5^. In the 1950s, the 5-year overall survival (OS) rate of patients with osteosarcoma was 22%^6^, but it has increased to 55–70% owing to the advancements in medicine in recent years^1,3,7–9^.

The Surveillance, Epidemiology, and End Results (SEER) program, sponsored by the National Cancer Institute (NCI), is a system of population-based cancer registries that currently covers approximately 28% of the US population from geographically defined areas^10^. Survival prediction models for osteosarcoma patients have been constructed previously^11–13^. However, the results of these studies have not been very satisfactory and they did not use data from the SEER database. Hence, further studies for better prediction models are needed.

For preparing prediction models for cancer, artificial intelligence (AI) models—constructed by machine learning (ML) algorithms—are common. However, most models are based on traditional ML algorithms created in the last century, including back propagation neural network (BPNN), multi-layer perceptron (MLP), decision tree, support vector machine (SVM), and Bayesian network^14^.

Extreme gradient boosting (XGBoost) is a large-scale machine-learning algorithm that was first officially published in 2016^15^. It is an improvement over the gradient boosting decision tree (GBDT). A single decision tree is a simple and weak classifier, but a tree ensemble model could be much better, such as the random forest^16^ and GBDT^17^. XGBoost is constructed by iterations for minimizing the loss of function^15^. Compared with GBDT, XGBoost uses a technique called ‘feature sub-sampling’, which is used in random forest to prevent over-fitting^15^. The XGBoost algorithm has been used widely in industries but rarely in medical research. Compared with traditional ML algorithms, XGBoost is more novel and complex. An important advantage of XGBoost over traditional ML algorithms is having random seeds that make the model better by repetitive operation even if the parameters are not changed. On comparison, SVM is not good at dealing with a problem with many samples and variables^18^, and the Bayesian network is easily and quickly trained, but is not complex enough.

Therefore, in the current study, we built an AI classifier by using the XGBoost algorithm to predict the 5-year survival of osteosarcoma patients, and aimed to construct a better AI prediction model. We extracted the samples from the SEER program database to train and cross-validate our prediction model. Additionally, to compare XGBoost to other traditional ML algorithms, we also built two other models by using SVM and the Bayesian network, which are common and representative ML algorithms in medical research. Receiver operating characteristic (ROC) analysis, area under the ROC curve (AUC) and decision curve analysis (DCA) of cross-validation were used for the evaluation of these three different models.

## Results

### Characteristics of the study population

**Figure 1:**
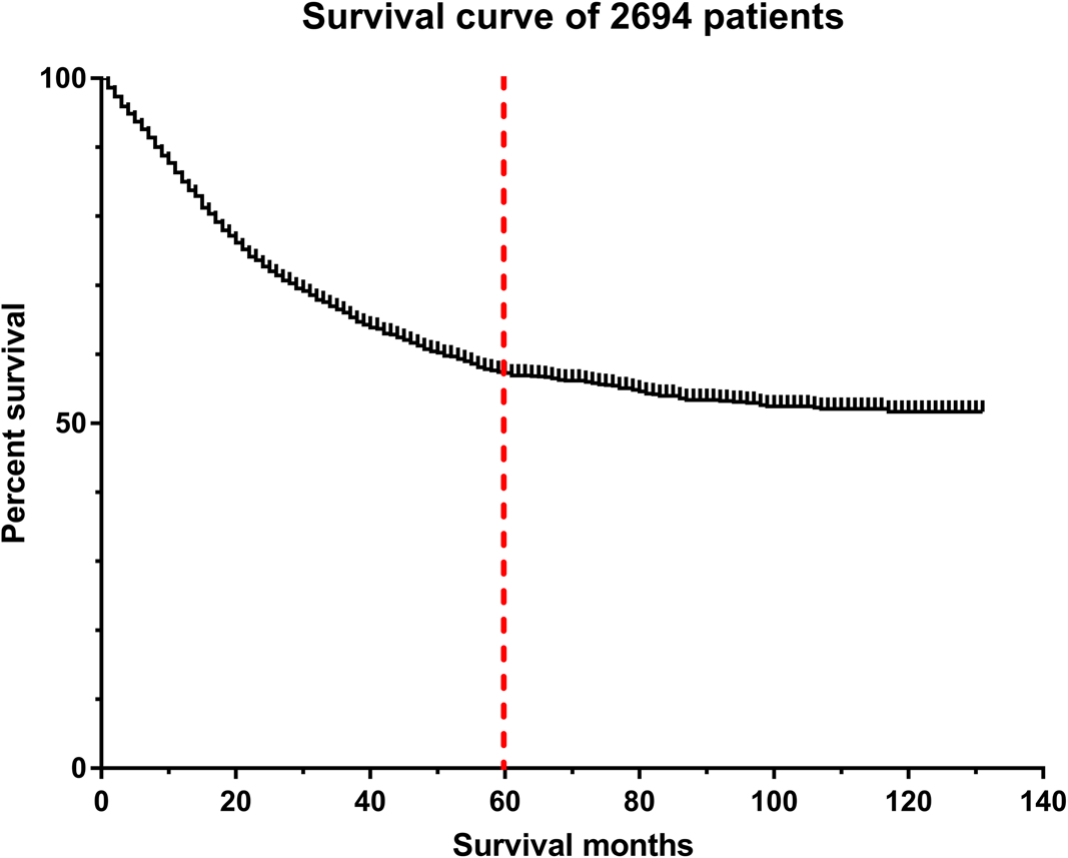
Overall survival curve for the 2694 osteosarcoma patients from the Surveillance, Epidemiology, and End Results (SEER) program database (2004–2014). The red line is the 5-year cut-off. The figure was created by using GraphPad Prism 7 (https://www.graphpad.com/).

**Figure 2:**
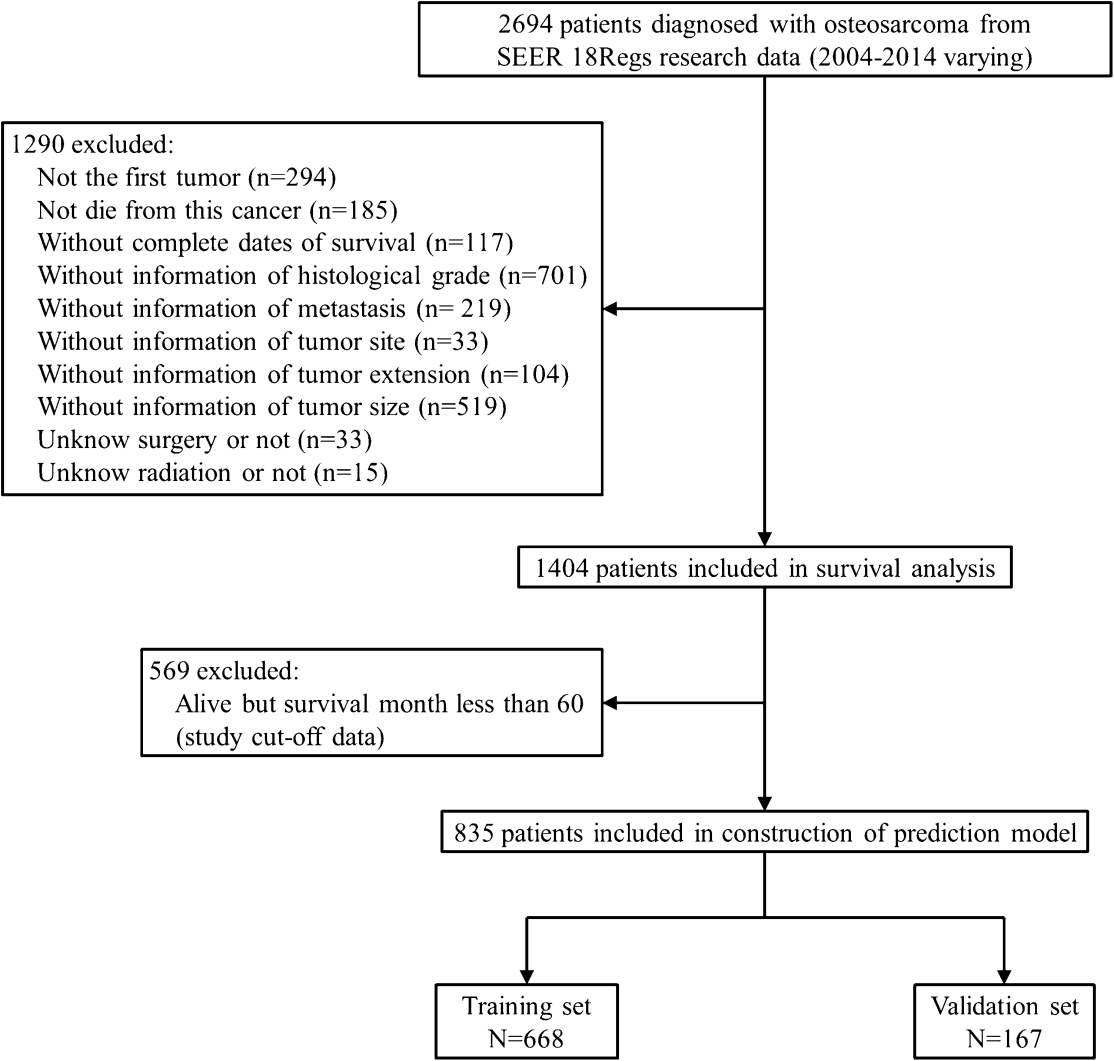
Flow chart showing the inclusion and exclusion process of patients in our study. The figure was created by using GraphPad Prism 7 (https://www.graphpad.com/).

The overall survival curve for 2694 osteosarcoma patients from the SEER program database declined much rapidly before the 5-year cut-off, compared with a slow downward trend in patient survival after 5 years (Fig. 1). Thus, predicting 5-year survival of osteosarcoma patients is of clinical value for treatment planning systems. We performed exclusion as shown in the flow chart (Fig. 2). Finally, 835 patients were included in our study. The study population was randomly divided into a training set (n = 668; 80%) and a validation set (n = 167; 20%).

There was no significant difference between the training and validation sets considering the 15 characteristics except primary tumor number (Table 1). The most common primary tumor sites were the arms and legs i.e., limbs (81.89% and 76.05% in the training and validation sets, respectively); few patients had local lymphatic metastasis (2.99% in the training and validation sets, both). Patients were more likely to have distant metastasis (21.21% and 19.76% in the training and validation sets, respectively). Most patients underwent surgery at the anatomical location (90.57% in the training set and 88.62% in the validation set) (Table 1).

**Table 1:** Characteristics of 835 patients included in the study.

Characteristics	Training set (N = 668)	Test set (N = 167)	*P*-value
**Sex**			0.807
Male	377 (56.44%)	96 (57.49%)	
Female	291(43.56%)	71 (42.51%)	
**Vital status**			0.466
Alive	355 (53.14%)	94 (56.29%)	
Dead	313 (46.86%)	73 (43.71%)	
Survival month	60.61 ± 39.34	62.28 ± 40.56	0.756
Age at diagnosis	27.66 ± 19.49	29.19 ± 20.65	0.687
Year of diagnosis	2007.30 ± 2.36	2007.37 ± 2.36	0.759
**Anatomical location**			0.069
Head and neck	55 (8.23%)	13 (7.78%)	
Trunk	66 (9.88%)	27 (16.17%)	
Limb	547 (81.89%)	127 (76.05%)	
**Histological grade**			0.650
Grade I	21 (3.14%)	7 (4.19%)	
Grade II	50 (7.48%)	16 (9.58%)	
Grade III	191 (28.60%)	48 (28.74%)	
Grade IV	406 (60.78%)	96 57.49%)	
**Extension**			0.914
Distant	155 (23.20%)	39 (23.35%)	
Localize	194 (29.04%)	51 (30.54%)	
Regional	319 (47.76%)	77 (46.11%)	
Tumor size (mm)	101.71 ± 61.78	93.86 ± 53.61	0.071
**Primary tumor number**			0.015
One primary only	637 (95.36%)	166 (99.40%)	
More primaries	31 (4.64%)	1 (0.60%)	
**Local lymphatic metastasis**			1.000
Yes	20 (2.99%)	5 (2.99%)	
No	648 (97.01%)	162 (97.01%)	
**Distant metastasis**			0.897
Yes	135 (20.21%)	33 (19.76%)	
No	533 (79.79%)	134 (80.24%)	
**Surgery**			0.450
Yes	605 (90.57%)	148 (88.62%)	
No	63 (9.43%)	19 (11.38%)	
**Radiation**			0.588
Yes	59 (8.83%)	17 (10.18%)	
No/unknown	609 (91.17%)	150 (89.82%)	
**Chemotherapy**			1.000
Yes	560 (83.83%)	140 (83.83%)	
No/unknown	108 (16.17%)	27 (16.17%)	

Continuous variates are reported as mean ± SD (standard deviation); classification variates are reported as numbers and percentage.

### Survival analyses

**Figure 3:**
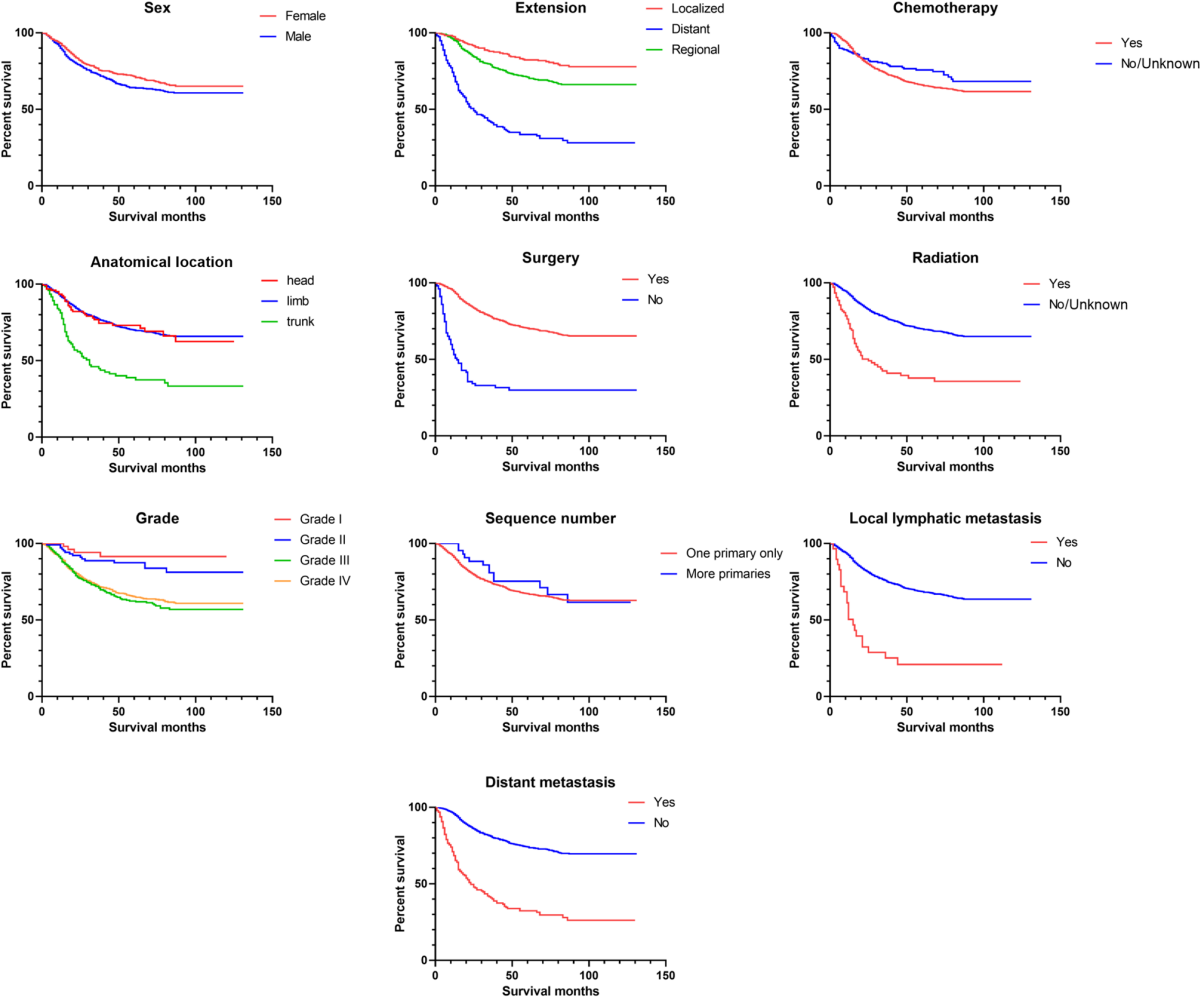
Kaplan–Meier survival curves to evaluate the influence of the ten classified characteristics (sex, anatomical location, histological grade, radiation, chemotherapy, tumor extension, primary tumor number, local lymphatic metastasis, distant metastasis, and surgery) for osteosarcoma patient survival. Anatomical location, site of the primary tumor. The figure was created by using GraphPad Prism 7 (https://www.graphpad.com/).

According to the Kaplan–Meier survival curves (Fig. 3) and log-rank tests for categorical variables, sex (P = 0.060), chemotherapy (P = 296) and primary tumor number (P = 0.500) were not significant factors influencing survival, but anatomical location (P < 0.001), histological grade (P = 0.001), tumor extension (P < 0.001), radiation (P < 0.001), local lymphatic metastasis (P < 0.001), distant metastasis (P < 0.001) and surgery (P < 0.001) significantly affected patient survival (Table 2). In the Cox proportional hazards regression model, the hazard ratio (HR) was used for evaluating the relationship between the corresponding variable and patient survival. Age (HR 1.682, 95% CI 1.538–1.840; P < 0.001) and tumor size (HR 1.266, 95% CI 1.185–1.353; P < 0.001) were significantly related to patient survival (Table 2). But year of diagnosis was not (HR 1.010, 95% CI 0.895–1.141; P = 0.867).

**Table 2:** Survival analysis (Log-rank test and Cox regression model) evaluating the influence of characteristics for osteosarcoma patient survival.

Characteristic	${\chi}^{2}$	*P*-value
Sex	3.541	0.060
Anatomical location	65.177	< 0.001
Histological grade	13.155	0.001
Primary tumor number	0.455	0.500
Local lymphatic metastasis	60.638	< 0.001
Distant metastasis	223.389	< 0.001
Extension	248.116	< 0.001
Radiation	67.922	< 0.001
Chemotherapy	1.093	0.296
Surgery	141.786	< 0.001

	Hazard ratio (95%)	
Age at diagnosis	1.682 (1.538, 1.840)	< 0.001
Year of diagnosis	1.010 (0.895, 1.141)	0.867
Tumor size	1.266 (1.185, 1.353)	< 0.001

We selected following characteristics into model construction: anatomical location, histological grade, tumor extension, radiation, local lymphatic metastasis, distant metastasis, surgery, age and tumor size. These characteristics were significantly in the survival analyses. In addition, we take chemotherapy into our model as it is an important predictor of survival.

### Model evaluation

**Figure 4:**
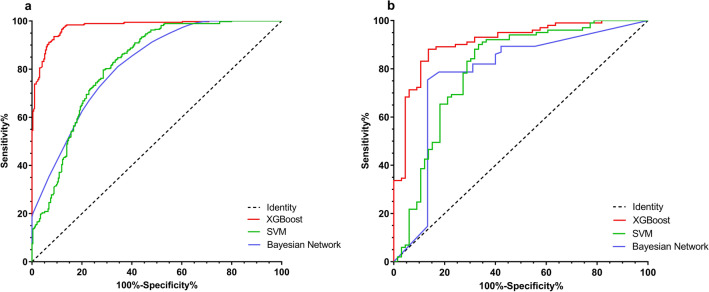
Receiver operating characteristic curves showing the predictions of the three models: XGBoost, SVM, and the Bayesian network. (**a**) The training set, (**b**) the validation set. XGBoost, extreme gradient boosting; SVM, support vector machine. The figure was created by using GraphPad Prism 7 (https://www.graphpad.com/).

Our model was tested and adjusted repeatedly, and the parameters were confirmed for the best model. The details of our model are shown in the supplementary materials (Material S1). To determine the accuracy of our models, we performed cross-validations. The ROC curves of the predictions for the training set (n = 668) and the validation set (n = 167) were constructed and the corresponding AUC was calculated. The XGBoost model had a better performance in the training set (AUC = 0.977, 95% confidence interval [CI] 0.968–0.986), compared with SVM (AUC = 0.817, 95% CI 0.785–0.852) and the Bayesian network (AUC = 0.817, 95% CI 0.785–0.849) (Fig. 4a.). In the validation set, the accuracy of the XGBoost model for predicting survival was higher (AUC = 0.911, 95% CI 0.865–0.956) than SVM (AUC = 0.801, 95% CI 0.726–0.876) and the Bayesian network (AUC = 0.781, 95% CI 0.689–0.873) (Fig. 4b). Our XGBoost model was better in predicting the 5-year survival of osteosarcoma patients as the AUC was over 0.9 in cross-validation (in both sets), compared to the other models.

**Figure 5:**
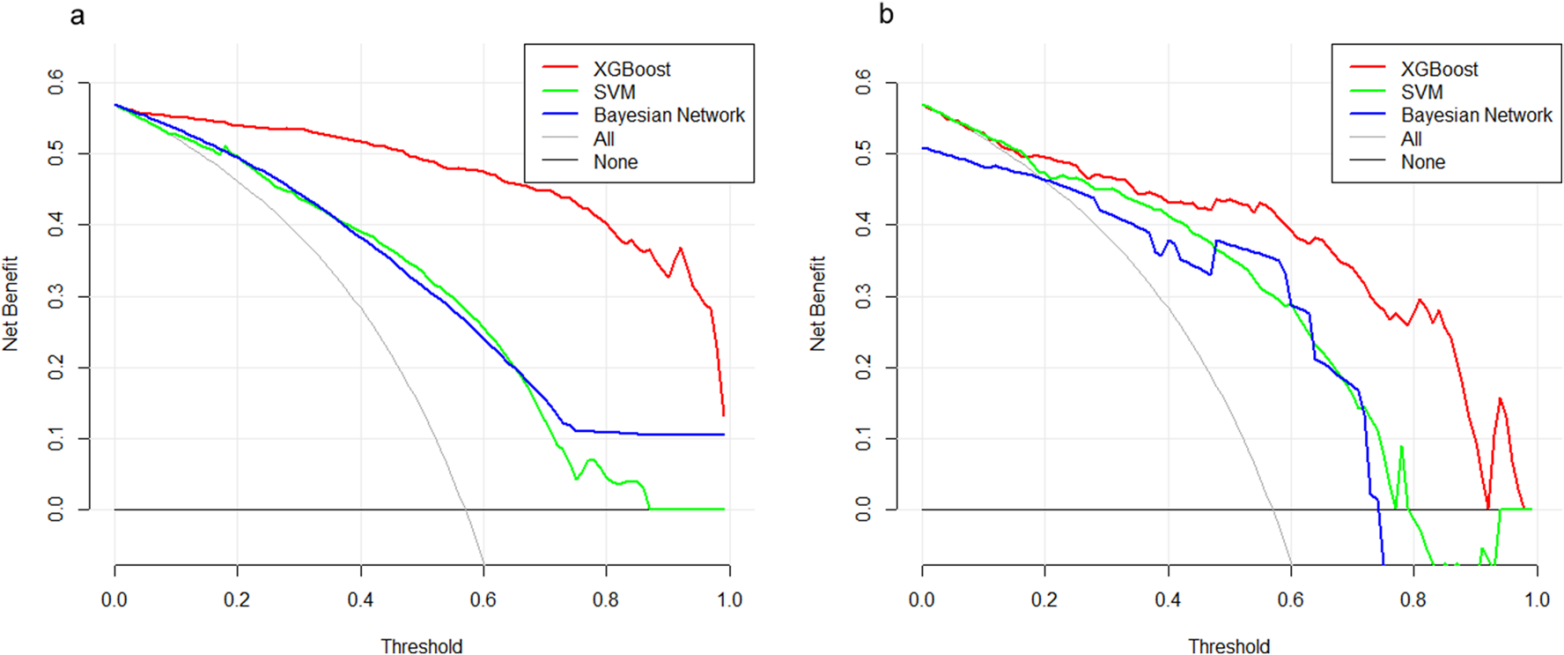
Decision curve analysis graph showing the net benefit against threshold probabilities based on decisions from model outputs. Three curves were based on predictions of the three different models, and the two curves were based on two kinds of extreme decisions. The curve called ‘All’ represents the prediction that all the patients would survive for 5 years, and the curve called ‘None’ represents the prediction that all the patients were dead at 5 years. (**a**) The training set, (**b**) the validation set. XGBoost, extreme gradient boosting; SVM, support vector machine. The figure was created by using R Version 3.4.4 (https://www.r-project.org/).

Decision curves of the three models were constructed in our study (Fig. 5). The y-axis of the decision curve represents the net benefit, a decision analytic measure judging whether clinical decisions have more benefit than harm. Each point on the x-axis represents a threshold probability that differentiates between patients with 5-year survival and those without. The decision curve of XGBoost was greater than that of the other two models because the net benefit was the highest for most of the thresholds.

## Discussion

Survival prediction for patients with malignancy is usually difficult but important, as it influences treatment planning and patient decision^19^. Compared with the empirical prediction from clinicians, our prediction model gives a more reliable choice for predicting the 5-year survival status of osteosarcoma patients. When clinicians prepare the plan for interventional or long-term therapy for patients, the expected survival time could be an influencing factor. Considering this, our prediction model could help prepare a reasonable therapy plan for personalized medicine.

Several survival prediction models have been used for osteosarcoma patients, including those based on nomograms (constructed by regression models)^13^, tomography images^12^, or the ML algorithm^11^. A 1-year survival prediction model using the Bayesian network was constructed in 2017^11^, with an AUC of 0.767. However, this was a single-center study. Moreover, the 1-year survival rate of osteosarcoma patients is much higher than 5-year survival rate (Fig. 1), and is therefore not as meaningful as the 5-year survival. Furthermore, a 5-year survival prediction model for predicting the survival of patents with high-grade osteosarcoma was prepared using radiomics of tomography images^12^. It was an innovative model, with an AUC of 0.86 in the training cohort and 0.84 in the validation cohort. However, this model used radiomics of tomography images to calculate a radiomics score for each patient and developed a multiple logistic regression model using radiomics score with the addition of several other characteristics. Logistic regression is a regular algorithm that can be replaced by a more complex algorithm. Thus, compared to these two studies, our study was a multicenter study and used a more accurate and stable algorithm to construct the prediction model. Therefore, our AI model based on XGBoost had a higher accuracy in predicting the 5-year survival of osteosarcoma patients (AUC = 0.977 and 0.911 in the training and validation sets, respectively); the accuracy of a prediction model is considered the most important quality^14^.

All the characteristics in our model were related to osteosarcoma patient prognosis. Histological grade and tumor extension influence survival time of patients. The histological grade of cancer is an indicator of the differentiation of tumor cells, and the tumor extension is used to express the degree of cancer progression^20,21^. Moreover, age, tumor site, metastasis, therapy, and tumor size are important prognostic factors for osteosarcoma patients^6,7,9^. In most previous prognostic models, age and tumor size were usually transformed to classified variables^11–13^. The use of the method for transforming variables could help calculate the risk for different kinds of patients and help list the risk in a table. In our prediction model, we preferred to calculate the 5-year survival probability of a specific patient. This gives a more detailed and personalized prediction, which provides medical plans as detailed and customized as possible rather than similar medical plans for a class of patients. Personalized medicine and precision medicine have been focus areas in recent years, both of which are based on large omics, molecular diagnostics, and high-throughput technologies^22–24^. Additionally, AI is an important tool for personalized medicine^25,26^, and our AI-based prediction model could help in personal therapy planning, thereby assisting in personalized medicine. For example, a clinician could not decide to recommend a patient to perform surgery or not. He could use our model with the variable “Surgery” as “yes” and “no”. Comparing the results given by the two conditions could help for his decision.

XGBoost has outstanding performance for processing large-scale and high-dimensional data^27^. However, for the first time, this algorithm has been used to construct prediction models for osteosarcoma patient survival. As XGBoost is good at dealing with complex problems, it is suitable for most other types of complex classification problems^27–29^.

Our study had some advantages. First, the SEER database provided complete information of patients covering widespread areas. Second, our AI model could provide personalized survival prediction for patients, thereby providing individualized therapy. Finally, our AI model can be used to determine survival for more osteosarcoma patients because all the information used for predicting survival is easily accessible and our model can be optimized as a software-based or web-based tool.

However, the study has some limitations. First, our study was retrospective; prospective randomized clinical trials will be needed to provide high-level evidence for clinical application. Second, we could not acquire the socioeconomic status, obviously related to patient survival, and the incidence of pathologic fractures, an important prognostic factor for osteosarcoma. Finally, in the SEER data, “no” and “unknown” combined in one category in chemotherapy and radiation. We could not ignore the underreporting of chemotherapy and radiation.

In conclusion, we used the XGBoost algorithm to construct an AI model predicting the 5-year survival of osteosarcoma patients. Age, primary tumor site, histological grade, tumor extension, tumor size, local lymphatic metastasis, distant metastasis, radiation, chemotherapy and surgery were the characteristics contributing to the model. Our AI prediction model had excellent accuracy according to ROC analyses. As the clinical value of the model was confirmed considering DCA, we believe the developed AI model could be used as a clinical tool for helping clinicians in making better treatment decisions for osteosarcoma patients^1^.

## Materials and methods

### Study population

We identified all cases of osteosarcoma listed in the SEER Research Database (2004–2014). The accession number is 10467-Nov 2018. There were 2694 cases and all were confirmed histologically as osteosarcoma. SEER*Stat Software (version 8.3.5) was used to extract these cases. We constructed a survival curve for the 2694 patients to evaluate the overall survival of osteosarcoma patients. However, most of the cases were excluded according to our inclusion and exclusion criteria. The inclusion criteria were as follows: (a) complete information about survival and follow-up available, (b) diagnosis of osteosarcoma as the primary malignant tumor. The exclusion criteria were as follows: (a) death due to other causes; (b) alive but survival < 5 years at the follow-up cut-off date; (c) information about tumor site, grade, tumor size, metastasis or therapy unavailable.

### Variable selection

After comprehensive analyses for prognostic factors of osteosarcoma considering our clinical knowledge and previous studies^7–9,30–33^, we selected 15 characteristics to be evaluated, including patient information (age, sex and year of diagnosis) and survival information (survival period and status at the follow-up cut-off date). Moreover, tumor information including the anatomical location, histological grade, tumor extension, tumor size, primary tumor number, local lymphatic metastasis, distant metastasis, radiation, chemotherapy and surgery was also taken into consideration.

We performed survival analyses using the patient and tumor information to determine the characteristics that significantly influenced patient survival. These analyses were performed before the exclusion of patients who alive but survival < 5 years at the follow-up cut-off date.

### Construction of the prediction model

Our prediction model was based on XGBoost, a scalable tree boosting system. The model was trained using the training set and tested using the validation set to determine model accuracy. Before running the training program, a response variable was obtained for survival information. It reflected the survival status of patients at 5 years, in which 1 = survival and 0 = death. One-hot encoding was performed for the three multi-classified variables (anatomical location, histological grade, and tumor extension). Normalization was performed for the two continuous variables (age and tumor size).

**Figure 6:**
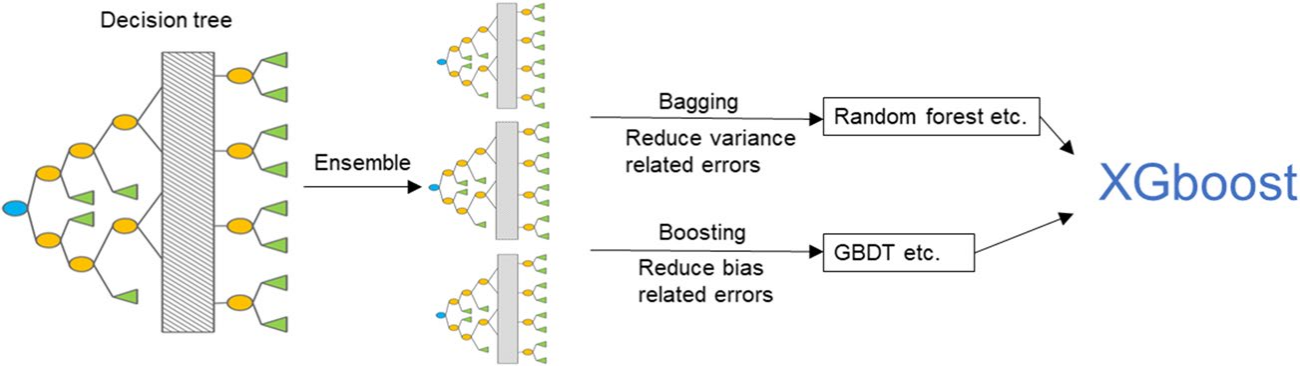
Schematic representation of the XGBoost model. XGBoost, extreme gradient boosting; GBDT, gradient boosting decision tree. The figure was created by using GraphPad Prism 7 (https://www.graphpad.com/).

Bagging (bootstrap aggregating) and boosting are ensemble learning methods that can integrate decision trees to reduce the model error^34^. XGBoost combines the advantages of these two methods and effectively reduces the bias-related error and variance-related error of the model (Fig. 6). In our prediction model, the number of ensemble decision trees was 30 and the maximum depth of each tree was 12. This was calculated via repeated tries to get the best accuracy and avoid overfitting. The outcomes of XGBoost were continuous outputs between 0 and 1, which represented the probability of the corresponding patient survival for > 5 years.

### Model evaluation

ROC curves were constructed for prediction in the training and validation sets. The AUC was used to evaluate the performance of our model. An AUC value between 0.5 and 1.0 is an important statistical property to evaluate binary classifiers^35^.

DCA that evaluates and compares prediction models incorporating clinical consequences was another way to evaluate our model^36^. Compared with traditional measures such as AUC that only represents the predictive accuracy, DCA give information about the clinical value of models^37^. In our study, decision curves were constructed to calculate the net benefit across different threshold probabilities of our prediction.

For comparing XGBoost with other ML classifiers, we constructed two other prediction models, respectively, based on SVM and the Bayesian network.

### Statistical analyses

The Mann–Whitney U test and chi-squared test were used to compare continuous variables and categorical variables, respectively. Kaplan–Meier survival analysis and log-rank test were performed to analyze the relationship between categorical variables and patient survival. A multivariate Cox proportional hazards regression model was constructed to analyze the relationship between continuous variables and patient survival. These test and analyses were performed using SPSS 25.0 software (IBM, Armonk, NY). R Version 3.4.4 (R Foundation for Statistical Computing, Vienna, Austria) was used to construct, train, and validate the prediction models with “xgboost” package. The decision curve analysis was also performed using R Version 3.4.4. A P-value of < 0.05 was considered statistically significant.

### Ethical considerations

We obtained permission to access the files of SEER database. The personal identifying information was not involved in this study so that the informed consent was not required. This study was reviewed and approved by the Medical Ethic Committee of Sir Run Run Shaw hospital affiliated to Medical College of Zhejiang University. And the study approval number is SRRSH2017092101.

### Ethical approval

Medical Ethic Committee of Sir Run Run Shaw hospital affiliated to Medical College of Zhejiang University waived the informed consent off because all the information of patients were accessed from SEER database (https://seer.cancer.gov/data/). We declare that all methods were performed in accordance with the relevant guidelines and regulations (Declaration of Helsinki).

## Supplementary Information

Supplementary Information.
