# Supplementary material for: Predictive model for the 5-year survival status of osteosarcoma patients based on the SEER database and XGBoost algorithm
Source: Sci Rep. 2021 Mar 10;11:5542. doi: 10.1038/s41598-021-85223-4 (PMC7970935; doi:10.1038/s41598-021-85223-4)
Supplement: Supplementary file 1 — Supplementary Information. [file 41598_2021_85223_MOESM1_ESM.docx]

**Predictive model for the 5-year survival status of osteosarcoma patients based on the SEER database and XGBoost algorithm**

Jiuzhou Jiang ^1,2,†^, Hao Pan ^3†^, Mobai Li ^1,2^, Bao Qian ^1,2^, Xianfeng Lin ^1,2,*^, Shunwu Fan ^1,2,*^

^1^ Department of Orthopaedic Surgery, Sir Run Run Shaw Hospital, Medical College of Zhejiang University, Hangzhou, China

^2^ Key Laboratory of Musculoskeletal System Degeneration and Regeneration Translational Research of Zhejiang Province, Hangzhou, China

^3^ Department of Orthopaedics, The First Affiliated Hospital of Wenzhou Medical University, Wenzhou, China

***** Correspondence: [xianfeng_lin@zju.edu.cn](mailto:xianfeng_lin@zju.edu.cn) (X.L.); [shunwu_fan@zju.edu.cn](mailto:shunwu_fan@zju.edu.cn) (S.F.)

**†** These authors have contributed equally to this work.

**Supplementary file:**

**Details of the XGBoost model in R Software**

##### xgb.Booster

raw: 228.1 Kb

call:

xgb.train(params = params, data = dtrain, nrounds = nrounds,

watchlist = watchlist, verbose = verbose, print_every_n = print_every_n,

early_stopping_rounds = early_stopping_rounds, maximize = maximize,

save_period = save_period, save_name = save_name, xgb_model = xgb_model,

callbacks = callbacks, eta = 0.05, max_depth = 12, subsample = 1,

colsample_bytree = 0.7, seed = 100, min_child_weight = 0.01,

eval_metric = "auc", base_score = 0.5, objective = "binary:logistic",

nthread = 5)

params (as set within xgb.train):

eta = "0.05", max_depth = "12", subsample = "1", colsample_bytree = "0.7", seed = "100", min_child_weight = "0.01", eval_metric = "auc", base_score = "0.5", objective = "binary:logistic", nthread = "5", silent = "1"

callbacks:

cb.print.evaluation(period = print_every_n)

cb.evaluation.log()

# of features: 17

niter: 30

nfeatures : 17

evaluation_log:

iter train_auc

1 0.890165

2 0.920445

---

29 0.975413

30 0.976841
